# Salivary pH, calcium, phosphorus and selected enzymes in healthy dogs: a pilot study

**DOI:** 10.1186/s12917-017-1256-4

**Published:** 2017-11-10

**Authors:** Ilaria Iacopetti, Anna Perazzi, Tamara Badon, Silvia Bedin, Barbara Contiero, Rebecca Ricci

**Affiliations:** Department of Animal Medicine, Production and Health, University of Padua, Legnaro (Padua), Italy

**Keywords:** Saliva, Biochemistry, Healthy dog

## Abstract

**Background:**

Saliva in dogs, as in humans, is a complex fluid secreted by different salivary glands in the oral cavity to protect the oral mucosa and teeth. The use of saliva as a substitute for blood in diagnosing and prognosticating disease in humans is widely accepted. Salivary biochemistry has also been used as a marker for periodontal disease in humans. No studies have as yet investigated the relation between salivary biochemistry and periodontal disease in dogs, however; neither has the salivary composition of healthy dogs with no oral disease been assessed. The purpose of this study was to obtain an overview on pH distribution and a set of salivary biochemical analytes (calcium, phosphorus, lactate dehydrogenase, lysozyme and amylase) commonly related to oral health in humans in a subset population of healthy young dogs with no periodontal disease or previous oral disease. Data were analyzed to gather salivary reference ranges for pH and each parameter and to assess a possible correlation between salivary and serum analytes.

**Results:**

Twenty-nine adult client-owned dogs were recruited for the study. Lactate dehydrogenase and lysozyme showed higher concentrations in saliva than in serum, whereas amylase showed the contrary. Salivary biochemistry values did not differ between males and females or between non-neutered and neutered individuals. No significant correlations between salivary and serum calcium, phosphorus, lactate dehydrogenase, amylase and lysozyme were identified in this study. Data allowed intervals for the salivary pH and other analytes investigated to be obtained from healthy dogs with healthy oral conditions.

**Conclusions:**

These preliminary data can contribute to enlarge our understanding of the functional role of saliva and its relation to oral health in dogs.

## Background

Saliva is a fluid mostly secreted from different salivary glands present in the oral cavity [1–3]. It contains a complex mixture of electrolytes and proteins with different biological roles in digestion, host defense, lubrication, and maintenance of oral and general health [1, 3, 4].

In human medicine, many researchers have used sialochemistry to diagnose systemic illnesses and monitor general patient health, and also as a disease risk indicator, demonstrating a close link between oral and systemic health [5]. There are compelling reasons to use saliva as a diagnostic fluid also in veterinary medicine. The greatest advantage is the method’s non-invasiveness: saliva is easily collectable from different animal species [6, 7]. In dogs, saliva samples have been mainly used for cortisol determination, avoiding the stress created by venipucture for blood collection [8], and also for IgGs measurement [9], rabies virus antigen detection [10], drug monitoring [11], and CRP quantification [1].

Some studies conducted on humans have identified different salivary biomarkers for oral health status, such as lactate dehydrogenase [12–14], mucin and amylase [4, 15], Il-1ß, MMP-8 and MMP-8/TIMP-1 ratios [16], lactoferrin [17, 18] lysozyme [19], and TNF-alpha [20]. These authors demonstrated that these analytes generally increase in proportion to periodontal disease in humans. Although periodontal disease is the most widespread oral disease in dogs, no studies have investigated the relationship between salivary biochemistry and the degree of severity of periodontal disease in this species [21]. Moreover, little is known on the salivary composition of healthy dogs with no oral disease.

Based on the limited literature available, the primary aim of this pilot study was to evaluate salivary pH and certain salivary analytes relevant to oral health, such as amylase, lactate dehydrogenase (LDH), lysozyme, calcium (Ca), and phosphorus (P) in healthy adult dogs with no oral disease. As these analytes have previously never been reported in literature, with exception of alpha-amylase, our results will eventually provide preliminary data to obtain salivary reference intervals. It is worth noting that as observed for certain analytes in humans, such as cortisol and adiponectin, saliva is a proven and accepted alternative to serum analysis in veterinary medicine [6]. A second objective is to assess whether parameter concentrations in saliva correlate with those found in serum.

## Methods

### Dogs

Adult client-owned dogs of different breeds and both sexes were recruited from patients of the Veterinary Teaching Hospital of the University of Padua. In order to be included in the study, dogs had to be young (less than 2 years old) and healthy on the basis of general physical examination, regularly vaccinated, and not to have received any antibacterial or anti-inflammatory medication for at least 6 months. Further inclusion criteria were no signs of oral pathology (grade 0 according to the classification of periodontal disease of the *American Veterinary Dental College*) [22] and no history of either oral disease or periodontal therapy, and dogs had to have been fed dry commercial food (kibbles). The study was approved by the Animal Welfare Committee of the University of Padua (authorization number n°#71/2015). The owner’s written consent was obtained before the dog was enrolled in the study.

### Saliva and blood collection

Both saliva and blood samples were obtained from all animals in the morning by the same examiner at the Veterinary Teaching Hospital of the University of Padua. In order to collect serum and saliva samples properly, dogs were prohibited from eating 12 h prior to sampling; furthermore, water was removed at least 1 h previously, as recommended by the manufacturer of the collection device. The oral cavity was thoroughly inspected to confirm the absence of any sign of oral pathology before saliva collection**.** Two dental cotton rolls^1^ were used, one at a time. Each one was inserted in the dog’s oral cavity using disposable gloves and pushed up to the molar teeth to make the dog chew for 1 min. Each cotton roll was immediately placed back in its own tube and centrifuged. After saliva collection, 6 ml of blood were taken by venipuncture from the cephalic vein of each dog using 22G needles and placed into evacuated plastic tubes containing either a coagulation accelerator or EDTA for biochemical and hematological analysis, respectively.

### Saliva and blood analysis

Saliva and blood samples were analyzed at the Clinical Diagnostic Laboratory of the Department of Animal Medicine Production and Health of the University of Padua.

Salivette® tubes were centrifuged^2^ for 2 min at 1000 g. After centrifugation, the two saliva samples obtained from each dog were pooled together in order to obtain a single sample of at least 300 μl of saliva. A visual quality assessment of hemolysis was assigned (using score 0 for no presence of blood to score 3 for severe presence of blood) to each saliva sample. Salivary pH was measured on each sample using litmus paper^3^. Saliva analysis, conducted using a BT1500 automated chemistry analyzer^4^, included the quantification of amylase, LDH, lysozyme, Ca, and P. The calibration of the equipment was the same used for blood.

The EDTA blood samples were analyzed using ADVIA 120 Hematology Systems – Siemens Healthcare^5^ equipped with Veterinary Software version 3.18.0-MS. A blood smear was performed for each sample to confirm the ADVIA data. The hematological analytes analyzed in this study included packed cell volume (PCV), platelet count (PLT), and leukocyte count (WBC), as well as the relative and absolute numbers of neutrophils, lymphocytes, monocytes, eosinophils, basophils, and large unstained cells (LUC) counts, and values for MPV, large platelet count, and platelet clumps (aggregates).

For serum biochemical analysis, coagulated samples were centrifuged (Labofuge 400, Heraeus Holding, Hanau, Germany) at 1750 g for 10 min at room temperature; serum was separated and immediately analyzed using a BT1500 automated chemistry analyzer. The analytes analyzed were: magnesium, alkaline phosphatase, aspartate aminotransferase, alanine aminotransferase, gamma-glutamyltransferase, LDH, creatinine, urea, total protein, globulins, cholesterol, triglycerides, glucose, and creatine kinase. Further, the analytes quantified in saliva were also measured in the serum. On board of the chemistry analyzer, Gesan reagents^6^ were used for all tests, except for lysozyme, for which the commercial kits^7^ , calibrated to manufacturer indications were used.

### Statistical analysis

The analytes analyzed in blood and saliva (amylase, LDH, lysozyme, Ca and P) were submitted to PROC UNIVARIATE (SAS V.9.1, SAS Institute Inc., Cary, North Carolina, USA) to test the normality of their distribution (Shapiro Wilk test ≥0.90), and all analytes that did not match the normal hypothesis were log-transformed. All normally distributed analytes were analyzed by a linear model with fixed effects of sex, sterilization, interaction between sex and sterilization (PROC GLM, SAS V.9.1, SAS Institute Inc., Cary, North Carolina, USA). Non-normally distributed analytes were analyzed by non-parametric analysis (Mann-Whitney *U-*test). Spearman’s test (PROC CORR, SAS V.9.1, SAS Institute Inc., Cary, North Carolina, USA) was used to assess the correlation between the analytes quantified in serum and saliva.

Reference intervals (lower and upper limits) of the salivary analytes measured in the study population were calculated on the basis of the recommendations for the determination of reference intervals in species of veterinary interest issued by the American Society for Veterinary Clinical Pathology (2011) using commercially available statistical MedCalc software^8^. In this analysis, the Robust method was adopted for the non-normally distributed salivary analytes, whereas the normal distribution method was used for the others. The Tukey method was used to identify suspected outliers, which were then excluded from analysis.

## Results

Twenty-nine (29) adult client-owned dogs met the inclusion criteria and were recruited for the study: 13 males (5 neutered) and 16 females (8 neutered), with a mean age of 18.2 months (range, 10 to 24) and a mean body weight of 16.5 kg (range, 6 to 42.8 kg). The breed population included 18 mixed breed dogs, 4 Flat Coat Retrievers, 2 Whippets, 1 American Staffordshire Terrier, 1 Breton, 1 Great Dane, 1 German Shepherd and 1 Basenji. All dogs had a complete set of teeth with periodontal disease grade 0 and no history of oral disease or periodontal therapy. Hematological and biochemical analyses revealed that the study population’s values were within the reference range (data not shown). All samples yielded a sufficient amount of saliva and could be used for analysis; none was discarded due to blood presence. The salivary pH and biochemistry values, as well as the same analytes measured in serum (mean ± DS, minimum, maximum and median), are shown in Table 1.

**Table 1:** Salivary and serum biochemistry values and salivary pH measured in the study population

Parameter	N	Mean (±DS)	Min	Max	Median	%95 CI for the mean
Saliva						
Ca mg/dl	29	7.48 ± 1,74	4.83	12	7.38	6.82–8.14
P mg/dl	29	3.57 ± 1,68	1.3	8.4	3.2	2.3–4.21
LDH UI/L	28	2394.9 ± 1669,5	341.2	7230.3	2013.1	1747.5–3042.2
Amylase UI/L	26	27.15 ± 19,18	6.25	95.66	25.48	19.40–34.90
Lysozyme mg/L	29	3.17 ± 4,08	0.25	20.03	1.77	1.61–4.72
pH	29	7.93 ± 0,46	7	9	8	7.76–8.10
Serum						
Ca mg/dl	28	10.35 ± 0,44	9.56	11.60	10.38	10.18–10.52
P _mg/dl_	28	4.67 ± 1,04	2.18	6.86	4.92	4.26–5.07
LDH UI/L	28	132.52 ± 79,71	43.65	362.38	106.24	101.61–163.43
Amylase UI/L	28	698.21 ± 223,29	325.34	1229.99	640.57	611.63–784.79
Lysozyme mg/L	28	0.36 ± 0,17	0.11	0.78	0.34	0.29–0.43

Min: lower identified value; Max: higher identified value

Some biochemistry analytes appear to be concentrated quite differently in saliva compared to serum: LDH and lysozyme are 18 and 9 times more concentrated in saliva, respectively, whereas amylase is 25 times less concentrated in saliva (Table 1); Ca and P presented a slightly higher concentration in serum than saliva (1.4 and 1.3 times, respectively). No significant effects of gender and reproductive state were detected for either saliva or serum analytes (Table 2).

**Table 2:** Means ± SE of serum and salivary biochemical analytes distributed according to gender and reproductive state

Parameter	Females	Males	Neutered	Non-neutered
Saliva				
Ca mg/dl	7.23 ± 0.36	8.00 ± 0.41	8.03 ± 0.42	7.20 ± 0.37
P mg/dl	3.38 ± 0.40	3.70 ± 0.45	3.37 ± 0.46	3.70 ± 0.40
LDH UI/L	2088.87 ± 1.22	1645.17 ± 1.27	1974.64 ± 1.26	1740.33 ± 1.24
Amylase UI/L	19.32 ± 1.19	24.72 ± 1.22	20.49 ± 1.22	23.31 ± 1.20
Lysozyme mg/L	1.88 ± 1.31	1.88 ± 1.36	2.81 ± 1.36	1.25 ± 1.31
Serum				
Ca mg/dl	10.45 ± 0.11	10.21 ± 0.14	10.41 ± 0.14	10.25 ± 0.11
P mg/dl	4.69 ± 0.24	4.77 ± 0.30	4.99 ± 0.31	4.47 ± 0.25
LDH UI/L	105.27 ± 1.16	130.01 ± 1.21	118.10 ± 1.21	115.89 ± 1.17
Amylase UI/L	657.90 ± 1.10	733.86 ± 1.12	624.19 ± 1.12	773.49 ± 1.10
Lysozyme mg/L	0.31 ± 1.11	0.34 ± 1.18	0.31 ± 1.18	0.34 ± 1.11

No significant correlations were found between salivary and serum Ca, P, LDH, amylase and lysozyme in this study (*p* > 0.05). The reference intervals for the salivary analytes measured in the 29 selected dogs, as well as the method used for their determination, are reported in Table 3.

**Table 3:** Reference intervals (lower and upper limits) of the salivary analytes measured in the study population based on the 90% CI of the mean (*P* values were calculated by the D’Agostino Pearson test; n SO: number of suspected outliers, detected by the Tukey method)

Analyte	P	n SO	Outliers	n dogs	Lower limit	Upper limit	Method
Ca mg/dl	0.03	2	11.99; 12	27	4.51	9.85	Robust
P mg/dl	0.014	1	8.4	28	0.15	6.27	Robust
LDH UI/L	0.005	3	33.8; 819.46; 7230	25	0	4025	Robust
Amylase UI/L	<0.001	1	95.66	25	0	52.05	Robust
Lysozyme mg/L	<0.001	2	12.21; 20.03	27	0	5.8	Robust
pH	0,09	0		29	7.03	8.82	Based on normal distribution

## Discussion

Saliva is a unique clear fluid, composed of electrolytes, immunoglobulins, proteins and enzymes mostly secreted from different salivary glands [3]. The basic role of saliva is to protect the oral mucosa and teeth through lubrication, buffering and clearance action, and antibacterial and antiviral activity, and is also involved in taste and digestion [3]. Its protective effect has prompted several studies on the characteristics (composition and pH) of human saliva in an attempt to establish a relationship with periodontal disease [2, 4, 23].

Despite the fact that periodontal disease is the most widespread oral disease in dogs, only very limited study on saliva composition and oral health biomarkers in particular has been conducted in veterinary medicine to date. To the authors’ knowledge, only one study has examined salivary pH and the concentration of certain minerals (e.g. Ca, P, Na and K) in healthy dogs, even if they play pivotal roles in tooth de/remineralization and calculus formation [7].

The primary aim of this study was therefore to evaluate a set of salivary biochemical analytes relevant to oral health such as amylase, LDH, and lysozyme, as well as calcium, phosphorus, and salivary pH in a population of healthy young adult dogs with no periodontal disease or history of oral disease. The objective was to obtain reference values for the biochemistry of saliva associated with oral health, given that none are as yet available in literature for this species. Differences between gender and reproductive state were also investigated, as well as correlations with the concentrations of the same analytes in serum.

In our study, average dog saliva pH was found to be slightly more acidic than as reported by Lavy et al. [7] (pH 8.53 ± 0.34, range 8.50–8.65), and we observed a wider range of values in the population tested (7–9, median 8). As in humans, it is reasonable to think that many factors affect oral pH in dogs. This lower average pH value than that obtained by Lavy et al. [7] could also be explained by different oral health conditions in the two populations investigated. Lavy et al. [7] did not provide any information on the oral health condition of the dogs included in his study, however, and therefore, whether some individuals included in his study suffered from periodontal disease or whether this may have affected salivary biochemistry or pH is unknown. Gender-related differences in saliva pH and biochemical profile have recently been observed in human medicine [24]. Female subjects have smaller salivary glands, and this may contribute to these differences. In our study, however, no significant differences between male and female dogs were detected, neither were any differences identified between non-neutered and neutered individuals. Further, we cannot exclude that canine breeds may affect salivary pH and future studies should focus on this topic, as this was not the aim of the present one. It is well known that salivary calcium and phosphate concentrations are involved in maintaining the balance between demineralization and remineralization [7, 25]. This makes them good candidates for the monitoring of oral health.

In our study, salivary concentrations of Ca (7.48 mg/dl) and P (3.57 mg/dl) were respectively lower and higher than the values reported by Lavy et al. [7] (Ca: 11.6 mg/dl; P: 1.3 mg/dl). More studies should be addressed to quantifying mineral concentrations in canine saliva, taking different breeds and ages, and different feeding management and oral conditions into consideration.

Lactate dehydrogenase (LDH) is a ubiquitous enzyme that plays a key role in the clinical diagnosis of many disease processes [13, 14, 26]. As it is released in the extracellular environment after cellular lysis, it represents a marker of tissue breakdown and cell death [27]. LDH activity has been therefore quantified in whole saliva and shown to be a reliable diagnostic marker of periodontal disease in humans [12, 23, 26]. In the study by De la Peña et al., [12] human patients with healthy periodontium showed significantly lower salivary LDH activity than those with periodontal disease (mean: 1107 vs 1930 UI/L, respectively). In our study, canine patients with healthy periodontium showed a salivary LDH activity comparable to that shown by De la Peña et al. [12] in human patients with periodontal disease (mean: 1930 UI/L). In humans, higher levels of salivary LDH have been attributed to cell death and tissue breakdown in the oral mucosa, and the study of salivary LDH isoenzyme patterns by De la Peña and colleagues indicates that the exfoliated cells of the oral epithelium are the major source of salivary LDH. [12, 26] It is therefore possible that the high LDH values observed in the healthy dog population in our study are the consequence of a para-physiologic increased cellular turnover linked to ethologic characteristics of this species. Dogs use their mouth not only to eat food but also as a means of social interaction with other animals and objects, and this increases the possibilities of oral mucosal lesions. Furthermore, De la Peña et al., [12] reported higher concentrations of LDH in females than in males, which were in accordance with previous studies in humans [28], whereas, as previously stated, no gender effect was detected in our canine population.

Lysozyme is an antibacterial enzyme, which is present in all body fluids, including saliva. In the oral cavity, lysozyme is secreted from major and minor salivary glands, gingival crevicular fluid, and salivary leukocytes [2, 23, 29]. It appears to play an important role in controlling microbial overgrowth, reducing the number of bacteria in the dental biofilm, decreasing colonization, and modifying bacterial metabolism [5, 30, 31]. The mean salivary concentration of lysozyme in our study (3.17 mg/L) was much lower than the level reported by Felizardo et al. [30] in healthy humans (41.74 to 93.86 mg/L).

α -Amylase is a family of proteins, consisting of several isoforms that differ in charge and glycosylation. It is secreted mainly by the parotid glands. This enzyme is considered a good indicator of properly functioning salivary glands [5]. Traditionally, this salivary enzyme was thought only to initiate the digestion of starch in the oral cavity, being responsible for the breaking down of high molecular weight carbohydrates to lower molecular weight sugars. In a study by Chauncey et al. on metabolic processes occurring within mammalian salivary glands, it was shown that dog’s parotid fluid is devoid of α-amylase activity and only traces were found in its submaxillary saliva. [32] This can be explained by the fact that dogs chew food very roughly, thus they do not need to initiate the digestion of starch in the mouth. However, in a recent study by Sousa-Pereira et al., a characterization of saliva in different mammal species was performed using a proteomic approach and α-amylase was detected in dogs’ saliva, which might reflect the natural selection related to digestion efficacy and food recognition. [33] Furthermore, Contreras-Aguilar et al. recently confirmed the existence of α -amylase in canine saliva and demonstrated that this enzyme can be measured by a spectrophotometric assay and that increases after a sympathetic activation. [34] From studies carried out in humans [4], it is now well-known that α-amylase is involved in the non-immunological defence mechanism of the oral cavity, and that salivary glands respond to the disease by increasing the protective potential of saliva and reducing the rate of secretion after the resolution of the inflammatory process. That study showed that people with no periodontitis have an average α -amylase activity of 93.90 U/ml [4], which is much higher than the mean value detected in the healthy canine population investigated in our study (27,15 U/L).

Saliva is a hypotonic fluid compared to plasma, and some components are found in lower, higher or similar concentration. [35] The measurement of selected analytes in saliva is a proven and accepted alternative to serum analysis in human medicine, and some analytes such as cortisol and adiponectin have been shown to correlate well with the blood concentration in dogs [6]. In our study, however, none of the analytes measured in saliva correlated with those measured in serum. Moreover, the higher LDH and lysozyme concentration in saliva compared to serum may be related to the species and be linked to the dogs’ increased need for oral mucosal defence. Contrarily, the lower salivary α -amylase concentration compared to serum, although potentially involved in the non-immunological defence of the oral cavity, may reflect the negligible need of this enzyme in saliva to digest starch.

Finally, the low number of subjects used in this study may represent a limitation of the reference interval calculation and these results should be confirmed in the future with a larger number of dogs.

## Conclusion

Using a population of young healthy dogs with no signs of oral disease to gather reference intervals for salivary biochemical analytes related to periodontal disease in humans may be an important step forward in enlarging our understanding of saliva’s functional role and relation to oral health in dogs. This study provides an overview of salivary composition with regard to certain biochemical analytes. No significant correlations between salivary and serum calcium, phosphorus, LDH, amylase and lysozyme were identified in this study. Conversely, LDH and lysozyme showed higher concentrations in saliva than in serum, whereas amylase was more concentrated in serum. Due to the paucity of information available in literature, however, further investigations on canine salivary biochemistry are recommended to confirm the results observed in this preliminary study and to investigate other analytes with possible associations to oral disease.

## Abbreviations

Ca: calcium
CRP: c reactive protein
EDTA: ethylenediaminetetraacetic acid
IgGs: immunoglobulins
Il-1ß: Interleukin
K: potassium
LDH: lactate dehydrogenase
LUC: large unstained cells
MMP: matrix metalloproteinase
MPV: mean platelet volume
Na: sodium
P: phosphorous
PCV: packed cell volume
PLT: platelets
TIMP-1: Tissue Inhibitor of Metalloproteinase
TNF-alpha: tumor necrosis factor alpha
WBC: white blood cells

## Footnotes

1 Salivette®, Sarstedt AG & Co, Nümbrecht, Germany

2 Labofuge 400, Heraeus Holding, Hanau, Germany

3 Carlo Erba Reagenti – Gruppo Montedison, Milano, Italy.

4 BT1500 automated chemistry analyzer, Biotecnica, Rome, Italy.

5 ADVIA 120 Hematology Systems, Siemens Healthcare, Milan, Italy.

6 Gesan Production s.r.l., Campobello di Mazara, Italy.

7 PKL, Paramedical s.r.l., Faiano, Italy.

8 MedCalc for Windows, version 12.5, MedCalc Software, Ostend, Belgium.
